# Pressure Effect on Elastic Constants and Related Properties of Ti_3_Al Intermetallic Compound: A First-Principles Study

**DOI:** 10.3390/ma11102015

**Published:** 2018-10-17

**Authors:** Xianshi Zeng, Rufang Peng, Yanlin Yu, Zuofu Hu, Yufeng Wen, Lin Song

**Affiliations:** 1 Research Center of Laser Fusion, China Academy of Engineering Physics, Mianyang 621900, China; zengxueliang@163.com; 2 State Key Laboratory for Environment-Friendly Energy Materials, Southwest University of Science and Technology, Mianyang 621010, China; rfpeng2006@163.com; 3 School of Mathematical Sciences and Physics, Jinggangshan University, Ji’an 343009, China; yuyanlin_121@163.com (Y.Y.); huzuofu@outlook.com (Z.H.); 4 State Key Laboratory of Solidification Processing, Northwestern Polytechnical University, Xi’an 710072, China; songlin@nwpu.edu.cn

**Keywords:** Ti_3_Al intermetallic compound, elastic properties, high pressure, first-principles calculations

## Abstract

Using first-principles calculations based on density functional theory, the elastic constants and some of the related physical quantities, such as the bulk, shear, and Young’s moduli, Poisson’s ratio, anisotropic factor, acoustic velocity, minimum thermal conductivity, and Debye temperature, are reported in this paper for the hexagonal intermetallic compound Ti${}_{3}$Al. The obtained results are well consistent with the available experimental and theoretical data. The effect of pressure on all studied parameters was investigated. By the mechanical stability criteria under isotropic pressure, it is predicted that the compound is mechanically unstable at pressures above 71.4 GPa. Its ductility, anisotropy, and Debye temperature are enhanced with pressure.

## 1. Introduction

Elastic constants are very important quantities to describe the mechanical properties of materials. They are evidently and directly employed to evaluate the elastic strains or energies in materials under stress of various origins: external, internal, thermal, etc. The plastic properties of materials are closely correlated with the shear moduli along the slip planes of mobile dislocations since these dislocations can dissociate into partials with a spacing determined by the balance between the planar fault energy and the repulsive elastic force [1]. Values of elastic constants provide valuable information on the structural stability, the bonding characteristic between adjacent atomic planes, and the anisotropic character of the bonding [1]. Elastic properties are also closely associated with many fundamental solid-state properties, such as acoustic velocity, thermal conductivity, Debye temperature, interatomic potentials, and so on.

The intermetallic titanium aluminides Ti${}_{3}$Al and TiAl are promising high-temperature structural materials for engine and airframe applications due to their low density and excellent high-temperature properties [2]. However, one main technological problem is to improve the room-temperature ductility of Ti${}_{3}$Al and TiAl for their practical use. It is well known that the crystal structure of a material plays a crucial role in determining its ductility. The ductility of the Ti${}_{3}$Al intermetallic compound is mainly attributed to the hexagonal crystal structure of the Ni${}_{3}$Sn-type DO${}_{19}$ with a limited number of available activated slip systems and the covalent bonding between the constituent elements. The slip on the (100) prism plane can be activated in DO${}_{19}$ Ti${}_{3}$Al while the slip on the (001) basal plane is seldom observed due to a high critical resolved shear stress [3]. On the other hand, alloys based on the TiAl intermetallic compound with a good balance of higher strength and better ductility generally possess dual-phase lamellar structures composed mainly of the L1${}_{0}$-ordered tetragonal TiAl (γ) phase and a small amount of the hexagonal Ti${}_{3}$Al ($\alpha_{2}$) phase [4]. The anisotropy of the deformation mode of the $\alpha_{2}$ phase strongly affects the plastic behavior of dual-phase alloys and, thus, the control of the plastic anisotropy of the $\alpha_{2}$ phase holds the key to improving the ductility of the alloys [5]. The growing interest in dual-phase alloys further encourages our interest in the intermetallic compound Ti${}_{3}$Al.

It is known that pressure is a key variable to tune a material’s solid-state properties. Nevertheless, only a few studies on the properties of Ti${}_{3}$Al under pressure have been carried out, to the best of our knowledge. Using the technique of high-pressure X-ray diffraction, Sahu et al. revealed that pressure in the range of 10–15 GPa induces the original Ni${}_{3}$Sn-type DO${}_{19}$ structure of Ti${}_{3}$Al to transform into the Ni${}_{3}$Ti-type DO${}_{24}$ one [6]. Subsequently, Rajaopalan et al. confirmed the pressure-induced phase transition in Ti${}_{3}$Al using first-principles calculations with the tight-binding linear muffin-tin orbital (TB-LMTO) method [7]. However, Dubrovinskaia et al. investigated in detail the behavior of Ti${}_{3}$Al with various Al content at pressures up to 25 GPa by means of high-pressure in situ powder X-ray diffraction and first-principles calculations with linear muffin-tin orbital atomic-sphere (LMTO-ASA) and full-potential linear muffin-tin orbital (FP-LMTO) methods [8]. They revealed there was no pressure-induced structural phase transition in Ti${}_{3}$Al up to 25 GPa. Thereafter, Zhang et al. studied the structural, elastic, and thermodynamic properties of Ti${}_{3}$Al under pressure up to 30 GPa using first-principles calculations based on density functional theory (DFT) and ultrasoft pseudo-potential (USPP) methods [9]. They found that the hexagonal structure of Ti${}_{3}$Al is elastically stable in the pressure range of 0–30 GPa, and the pressure can improve its ductility.

To gain a deeper understanding of the high-pressure properties for Ti${}_{3}$Al, in the present work, we focus on studying the elastic constants and some properties related to these constants for this compound in the range of 0–80 GPa by first-principles calculations based on DFT and projector augmented wave (PAW) methods.

## 2. Theoretical Methods

### 2.1. Computational Details

All the calculations based on DFT were carried out using the Vienna Ab initio Simulation Package (VASP, version 5.4) [10,11,12]. The ion–electron interaction is described by the PAW method [13,14]. The exchange-correlation functional is treated with the Perdew, Burke, and Ernzerhof (PBE) generalized gradient approximation (GGA) [15,16]. The valence electron configurations for Ti and Al correspond to 3s${}^{2}$3p${}^{6}$3d${}^{2}$4s${}^{2}$ and 3s${}^{2}$3p${}^{1}$. The plane wave cut-off energy was set as 600 eV. A convergence criterion of $10^{-6}$ eV/atom was used for the electronic self-consistency loop. The *k*-point meshes for the Brillouin zone sampling was constructed through the Monkhorst-Pack scheme [17]. An 11×11×13 *k*-points grid was used. Before calculating the elastic constants of the Ti${}_{3}$Al intermetallic compound at a given pressure *P*, the unit cell of the compound at the corresponding pressure was optimized by full relaxation with respect to the volume, shape, and internal atomic positions until the atomic forces were less than $10^{-2}$ eV/Å.

### 2.2. Calculations of Elastic Constants

The number of independent single-crystal elastic constants is five for the hexagonal intermetallic compound Ti${}_{3}$Al, i.e., $C_{11}$, $C_{12}$, $C_{13}$, $C_{33}$, and $C_{44}$. Starting from the optimized unit cells under different pressures, in this study, we used the strain–stress relationship method to determine all five elastic constants, as implemented in the VASP [18]. The elastic constants are defined as the first derivatives of the stresses with respect to the strain tensor. The elastic tensor was determined by performing six finite distortions of the lattice and deriving the elastic constants from the strain–stress relationship [19]. The elastic tensor was calculated for rigid ions, as well as allowing for relaxation of the ions. The ionic contributions were determined by inverting the ionic Hessian matrix and multiplying with the internal strain tensor [20]. The final elastic constants include both the contributions from distortions with rigid ions and the contributions from the ionic relaxations.

## 3. Results and Discussion

### 3.1. Bulk Properties at Zero Pressure

**Figure 1:**
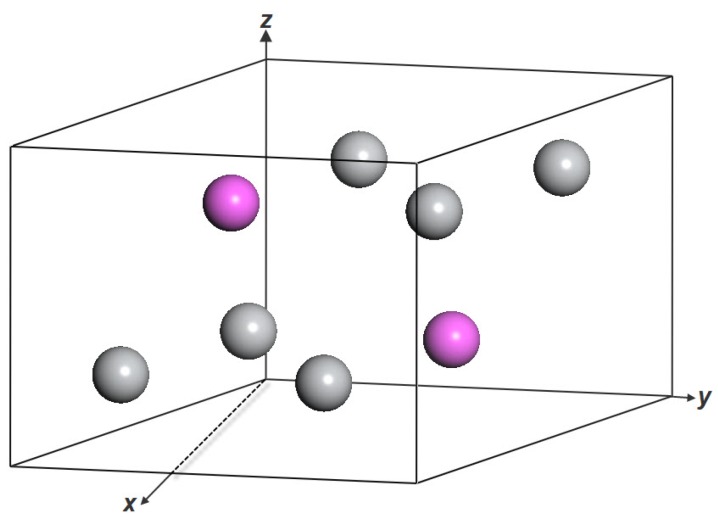
Crystal structure of Ti${}_{3}$Al.

The crystal structures of the hexagonal intermetallic compound Ti${}_{3}$Al are shown in Figure 1. There are a total of eight atoms in the unit cell of the compound. Among these atoms, six Ti atoms occupy 6 h Wyckoff positions and two Al atoms occupy 2c Wyckoff positions. Experimentally, the single-crystal elastic constants of Ti${}_{3}$Al have been measured by Tanaka and Koiwa [21] using the rectangular parallelepiped resonance method. Theoretically, the elastic properties of Ti${}_{3}$Al have been investigated by Zhang et al. [9], Wei et al. [22], and Liu et al. [23] with the USPP and the GGA methods, by Music and Schneider [24] with the PAW and the GGA methods, and by Fu et al. [25] with the full-potential linearized augmented plane-wave (FLAPW) and the local density approximation (LDA) methods. Table 1 presents the ground-state equilibrium lattice parameter and the elastic constants of the Ti${}_{3}$Al intermetallic compound in comparison with reported experimental data [21,26] and other theoretical values [9,22,23,24,25]. Comparison of the lattice parameters *a* and c/a shows that the present results are well consistent with the available experimental and theoretical results. The c/a ratio of 0.808 is correctly predicted in this study and fits well with the experimental value of 0.80. The presently obtained elastic constants are in good agreement with the experimental data [21], exhibiting smaller deviations as compared to the other theoretical results [9,22,23,24,25]. These indicate that the present calculation conditions are sufficiently reliable. Moreover, the mechanical stability conditions for hexagonal crystals at 0 GPa are as follows [27]:

(1) $$\begin{array}{c} C_{44}>0,\;\;C_{11}-\left|C_{12}\right|>0,\;\;C_{33}\left(C_{11}+C_{12}\right)-2C_{13}^{2}>0 \end{array}$$

These conditions are associated with different deformations of the crystals. A value approaching zero indicates the shear deformation of the cell for $C_{44}$, the expansion of the direction along the spindle axis during the contraction of the other symmetry direction perpendicular to the spindle axis for $C_{11}-\left|C_{12}\right|$, and the deformation of the volume for $C_{33}\left(C_{11}+C_{12}\right)-2C_{13}^{2}$. The elastic constants of Ti${}_{3}$Al obtained in this study satisfy the three conditions above, which shows that its hexagonal structure is mechanically stable at zero pressure.

### 3.2. Pressure-Dependent Structure Parameters

**Figure 2:**
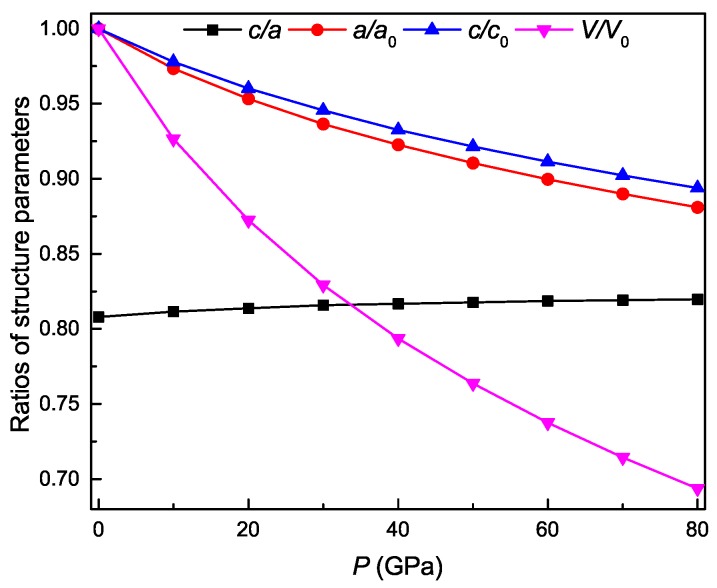
The ratios of structural parameters as a function of pressure for Ti${}_{3}$Al.

Figure 2 shows the pressure dependence of the lattice constant ratio *c*/*a*, the normalized lattice parameters *a*/$a_{0}$, *c*/$c_{0}$, and the normalized volume *V*/$V_{0}$ of Ti${}_{3}$Al, where $a_{0}$, $c_{0}$, and $V_{0}$ are the optimized lattice constants and volume at 0 GPa, respectively. The slight increase of the value for *c*/*a* with pressure in the figure indicates the better resistance to compression along the *c* axis. Meanwhile, the values of *a*/$a_{0}$, *c*/$c_{0}$, and *V*/$V_{0}$ monotonically decrease with pressure. The normalized parameter *a*/$a_{0}$ changes more rapidly than the counterpart *c*/$c_{0}$. At same pressure, the value of *a*/$a_{0}$ is always smaller than that of *c*/$c_{0}$. These also indicate the better resistance to compression along the *c* axis.

### 3.3. Pressure-Dependent Elastic Constants and Mechanical Properties

The calculated results of the elastic constant for Ti${}_{3}$Al under different pressures are presented in Table 2. The elastic constants $C_{11}$ and $C_{33}$ represent the elasticity in length. The other constants $C_{12}$, $C_{13}$, and $C_{44}$ are associated with the elasticity in shape. One can find that the four elastic constants for the hexagonal Ti${}_{3}$Al increase monotonically with pressure except for $C_{44}$. The constants $C_{11}$ and $C_{33}$ change rapidly with pressure, $C_{12}$ and $C_{13}$ change moderately under pressure, while $C_{44}$ incipiently increases slightly with pressure up to 40 GPa and then decreases gently under higher pressure. One can also find that the elastic constant $C_{11}$ is always smaller than the counterpart $C_{33}$ at the same pressure, showing that it is easier to compress along the [100] direction than along the [001] direction. Moreover, for hexagonal crystals, the conditions of mechanical stability under isotropic pressure are given by [28]

(2) $$\begin{array}{c} {\tilde{C}}_{44}>0,\;\;{\tilde{C}}_{11}-\left|{\tilde{C}}_{12}\right|>0,\;\;{\tilde{C}}_{33}\left({\tilde{C}}_{11}+{\tilde{C}}_{12}\right)-2{\tilde{C}}_{13}^{2}>0\;\; \end{array}$$

with

(3) $$\begin{array}{c} {\tilde{C}}_{ii}=C_{ii}-P\;\left(i=1,3,4\right),\;\;{\tilde{C}}_{12}=C_{12}+P,\;\;{\tilde{C}}_{13}=C_{13}+P \end{array}$$

**Figure 3:**
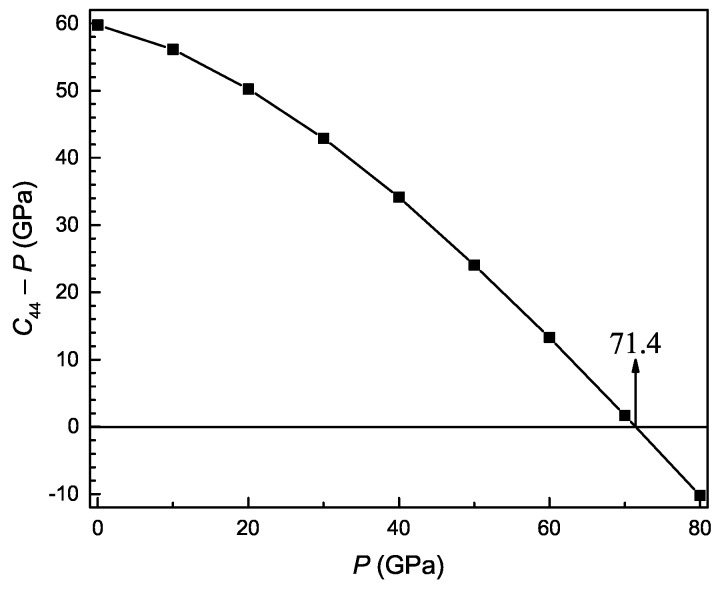
The constant $C_{44}-P$ as a function of pressure for Ti${}_{3}$Al.

The three conditions are related to different deformations of the crystals under isotropic pressure. A value approaching zero indicates the shear deformation of the cell under isotropic pressure for ${\tilde{C}}_{44}$, the expansion of the direction along the spindle axis under isotropic pressure during the contraction of the other symmetry direction perpendicular to the spindle axis under isotropic pressure for ${\tilde{C}}_{11}-\left|{\tilde{C}}_{12}\right|$, and the deformation of the volume under isotropic pressure for ${\tilde{C}}_{33}\left({\tilde{C}}_{11}+{\tilde{C}}_{12}\right)-2{\tilde{C}}_{13}^{2}$. Figure 3 shows the pressure dependence of ${\tilde{C}}_{44}$ for the Ti${}_{3}$Al intermetallic compound. When the value of ${\tilde{C}}_{44}$ is no longer larger than zero, it indicates that the hexagonal structure of the compound is mechanically unstable above pressures of about 71.4 GPa.

Sahu et al. [6] performed high-pressure X-ray diffraction studies of Ti${}_{3}$Al, and revealed a phase transition from the DO${}_{19}$ to DO${}_{24}$ structure in the pressure range of 10–15 GPa. Then, Rajaopalan et al. [7] theoretically confirmed the finding by taking the TB-LMTO approach within the atomic-sphere approximation (ASA) and the LDA. Dubrovinskaia et al. [8] pointed out that the high-pressure study in Ref. [6] was conducted on one single sample, and the interpretation of the results was not unambiguous, in addition to the fact that the calculations reported in Ref. [7] were conducted within the so-called ASA, which sometimes fails to resolve small structural energy differences. Thus, they performed a series of experiments on a number of samples with different compositions of Ti${}_{3}$Al by means of high-pressure in situ powder X-ray diffraction, and theoretically complemented them by using the LMTO-ASA method with the coherent potential approximation (CPA), and the FP-LMTO method with the GGA [8]. In their study, neither experiment nor theory observed the pressure-induced phase transition from the DO${}_{19}$ to the DO${}_{24}$ structure under pressure conditions of up to 25 GPa, and the possible reasons for the difference between their experimental study results and those of Sahu et al. [6] were also analyzed in detail. Zhang et al. [9] studied the structural stability of Ti${}_{3}$Al under pressures up to 30 GPa with the USPP and the GGA methods and found that the DO${}_{19}$ structure of Ti${}_{3}$Al is mechanically stable in the pressure range of 0–30 GPa. The result of our theoretical study agrees with that of the experimental and theoretical study in Ref. [8] and the theoretical study in Ref. [9].

From the obtained constants $C_{ij}$s of a single crystal, the bulk modulus (*B*) and shear modulus (*G*) of the polycrystal materials can be calculated by the Voigt–Reuss–Hill (VRH) approximation [29,30,31]. For the hexagonal structure, the bulk and shear moduli in the VRH approximation are given by

(4) $$\begin{array}{c} B=\frac{B_{V}+B_{R}}{2},\;\;\;\;G=\frac{G_{V}+G_{R}}{2} \end{array}$$

where $B_{V}$ and $G_{V}$ correspond to Voigt’s bulk modulus and shear modulus, $B_{R}$ and $G_{R}$ are Reuss’s bulk modulus and shear modulus, respectively, and they are given by [32,33]

(5) $$\begin{array}{c} B_{V}=\frac{2C_{11}+2C_{12}+4C_{13}+C_{33}}{9},\;\;G_{V}=\frac{C_{11}+C_{12}-4C_{13}+2C_{33}+12C_{44}+12C_{66}}{30},\\ B_{R}=\frac{\left(C_{11}+C_{12}\right)C_{33}-2C_{13}^{2}}{C_{11}+C_{12}-4C_{13}+2C_{33}},\;\;G_{R}=\frac{5\left[\left(C_{11}+C_{12}\right)C_{33}-2C_{13}^{2}\right]C_{44}C_{66}}{6B_{V}C_{44}C_{66}+2\left[\left(C_{11}+C_{12}\right)C_{33}-2C_{13}^{2}\right]\left(C_{44}+C_{66}\right)} \end{array}$$

with

(6) $$\begin{array}{c} C_{66}=\frac{C_{11}-C_{12}}{2} \end{array}$$

Further, the Young’s modulus (*E*) and Poisson’s ratio (ν) are estimated by [29,30,31]

(7) $$\begin{array}{c} E=\frac{9BG}{3B+G},\;\;\nu =\frac{3B-2G}{6B+2G} \end{array}$$

**Figure 4:**
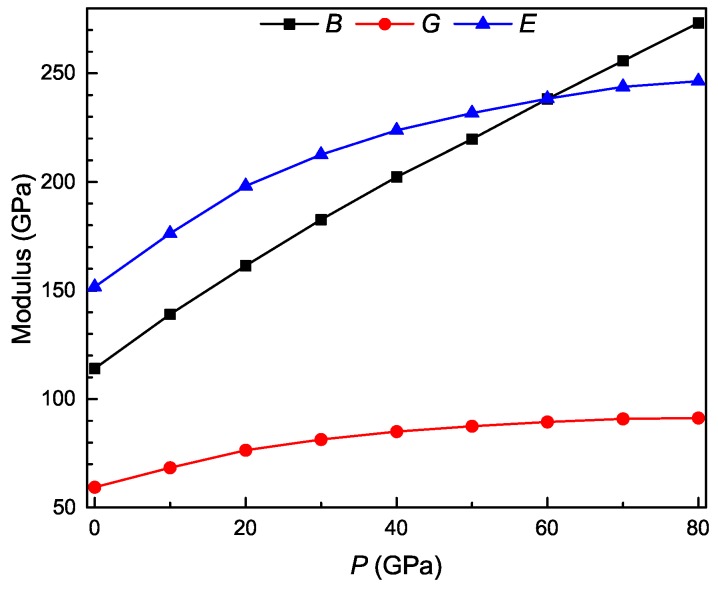
Bulk (*B*), shear (*G*), and Young’s (*E*) moduli as a function of pressure for Ti${}_{3}$Al.

Figure 4 shows the pressure dependence of bulk, shear, and Young’s moduli and Poisson’s ratio for the Ti${}_{3}$Al intermetallic compound. The bulk modulus reflects the resistance of materials against volume change. The shear modulus reflects the resistance of materials against shape change. The Young’s modulus measures the stiffness of materials, and the larger its value, the stiffer the material. From Figure 4, it is clearly seen that the values of *B*, *G*, and *E* increase monotonously with pressure, and the change trend is linear for *B* while nonlinear for *G* and *E*. These results mean that the volume change resistance increases linearly, while the shape change resistance and stiffness increase nonlinearly with pressure. Our calculated bulk, shear, and Young’s moduli at zero pressure are 114.04, 59.33, and 151.69 GPa, respectively, which are in excellent agreement with the corresponding experimental data of 113, 59, and 151 GPa [21]. Meanwhile, our calculated zero-pressure bulk modulus is also in excellent agreement with the theoretical values of 114 GPa [22] and 112.54 GPa [24].

**Figure 5:**
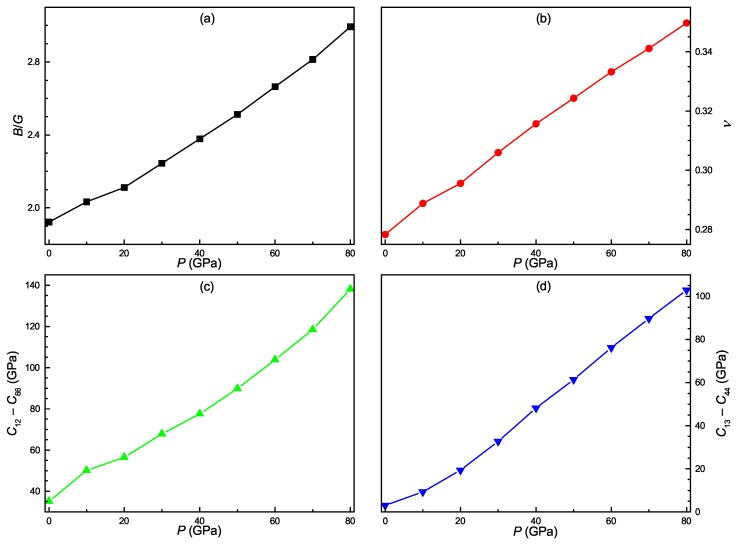
*B*/*G* ratio (**a**), Poisson’s ratio (**b**), and Cauchy pressures $C_{12}-C_{66}$ (**c**), $C_{13}-C_{44}$ (**d**) as a function of pressure for Ti${}_{3}$Al.

For the specific case of hexagonal crystals, the Cauchy pressure is defined as ($C_{13}-C_{44}$) for (100) plane and ($C_{12}-C_{66}$) for the (001) plane. Figure 5 shows the pressure dependence of the *B*/*G* ratio, Poisson’s ratio, and Cauchy pressures for the Ti${}_{3}$Al intermetallic compound. These quantities allow us to assess the ductility/brittleness of materials. According to Pugh’s rule [34], the high and low values of *B*/*G* are related to ductility and brittleness, respectively. Ductile behavior is exhibited in materials when B/G>1.75, otherwise the materials behave in a brittle manner. As shown in Figure 5a, the B/G ratio increases monotonically with pressure, which is always more than 1.75 in the studied pressure range. These indicate that Ti${}_{3}$Al becomes more ductile with pressure. According to the rule proposed by Frantsevich et al. [35], a brittle behavior is exhibited in materials when ν<0.26, otherwise the materials behave in a ductile manner. As shown in Figure 5b, Poisson’s ratio also increases monotonically with pressure, which is always more than 0.26 in the studied pressure range. These also indicate that Ti${}_{3}$Al becomes more ductile with pressure. According to Pettifor’s rule [36], the materials with larger positive Cauchy pressures have more metallic bonds and thus become more ductile, otherwise the Cauchy pressures of the materials are more negative, they have more angular bonds, and thus exhibit more brittleness. As shown in Figure 5c,d, the increase of positive values for both Cauchy pressures with pressure indicate that there is more metallic bonding in Ti${}_{3}$Al with pressure, and thus it becomes more ductile. At the same pressure, the Cauchy pressure ($C_{12}-C_{66}$) is always larger than the counterpart ($C_{13}-C_{44}$), implying that the metallic character of the bonding in the (001) plane is more significant than that in the (100) plane. Hardness is a measure of the resistance to elastic deformation, plastic deformation, or failure under external force; these are dependent on elastic constants, plasticity, strain, ductility, strength, etc. Theoretically, the hardness (*H*) of polycrystalline materials can be estimated by [37]

(8) $$\begin{array}{c} H=2{\left(\frac{G^{3}}{B^{2}}\right)}^{0.585}-3 \end{array}$$

**Figure 6:**
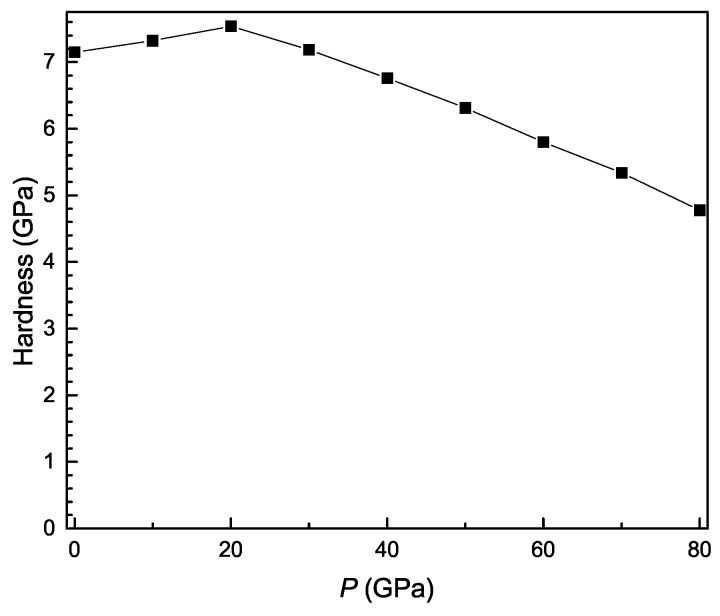
Hardness as a function of pressure for Ti${}_{3}$Al.

The pressure dependence of theoretical hardness is shown in Figure 6. It is clearly observed that the hardness decreases approximately with pressure.

### 3.4. Pressure-Dependent Elastic Anisotropy

**Figure 7:**
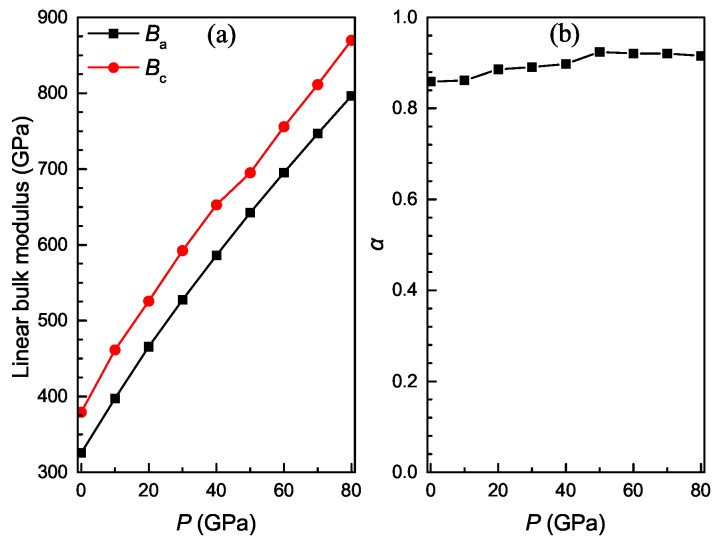
Linear bulk modulus along the a and c principle axes (**a**) and its anisotropy α (**b**) as a function of pressure for Ti${}_{3}$Al.

In material science, elastic anisotropy is the directional dependence of the physical properties of materials. Most materials exhibit elastically anisotropic behavior. Some examples are the directional dependence of the bulk modulus, Young’s modulus, shear modulus, and Poisson’s ratio. For hexagonal system, the linear bulk modulus along the a and c principle axes ($B_{a}$ and $B_{c}$) are given by [38]

(9) $$\begin{array}{c} B_{a}=a\frac{dP}{da}=\frac{\lambda }{2+\alpha },\;\;B_{c}=c\frac{dP}{dc}=\frac{B_{a}}{\alpha } \end{array}$$

with

(10) $$\begin{array}{c} \lambda =2\left(C_{11}+C_{12}\right)+4C_{13}\alpha +C_{33}\alpha^{2},\;\;\alpha =\frac{C_{11}+C_{12}-2C_{13}}{C_{33}-C_{13}} \end{array}$$

where α also signifies the anisotropy of linear compressibility along the *a* or *c* axis. For an isotropic material, the value of α must be unity. A deviation less than or greater than unity represents the degree of anisotropy. As shown in Figure 7b, Ti${}_{3}$Al presents elastic anisotropy due to its α being a value smaller than unity. The values of α increase approximately with pressure, but the variation is very small.

The average Young’s modulus on the (2$\bar{1}$0) and (010) prismatic planes and the (001) basal plane ($E_{\left(2\bar{1}0\right)}$, $E_{\left(010\right)}$, and $E_{\left(001\right)}$) are given by [30,39]

(11) $$\begin{array}{c} E_{\left(2\bar{1}0\right)}=E_{\left(010\right)}=\frac{1}{S_{11}},\;\;E_{\left(001\right)}=\frac{1}{S_{33}} \end{array}$$

with

(12) $$\begin{array}{c} S_{11}=\frac{1}{2}[\frac{C_{33}}{C_{33}\left(C_{11}+C_{12}\right)-2C_{13}^{2}}+\frac{1}{C_{11}-C_{12}}],\;\;S_{33}=\frac{C_{11}+C_{12}}{C_{33}\left(C_{11}+C_{12}\right)-2C_{13}^{2}} \end{array}$$

The average shear modulus on the (2$\bar{1}$0) and (010) prismatic planes and the (001) basal plane ($G_{\left(2\bar{1}0\right)}$, $G_{\left(010\right)}$, and $G_{\left(001\right)}$) are given by [30,39]

(13) $$\begin{array}{c} G_{\left(2\bar{1}0\right)}=G_{\left(010\right)}=\frac{2}{2\left(S_{11}-S_{12}\right)+S_{44}},\;\;G_{\left(001\right)}=\frac{1}{S_{44}} \end{array}$$

with

(14) $$\begin{array}{c} S_{11}-S_{12}=\frac{1}{C_{11}-C_{12}},\;\;S_{44}=\frac{1}{C_{44}} \end{array}$$

The average Poisson’s ratio on the (2$\bar{1}$0) and (010) prismatic planes and the (001) basal plane ($\nu_{\left(2\bar{1}0\right)}$, $\nu_{\left(010\right)}$, and $\nu_{\left(001\right)}$) are given by [30,39]

(15) $$\begin{array}{c} \nu_{\left(2\bar{1}0\right)}=\nu_{\left(010\right)}=-\frac{S_{12}+S_{13}}{2S_{11}},\;\;\nu_{\left(001\right)}=-\frac{S_{13}}{S_{33}} \end{array}$$

with

(16) $$\begin{array}{c} S_{11}=\frac{1}{2}[\frac{C_{33}}{C_{33}\left(C_{11}+C_{12}\right)-2C_{13}^{2}}+\frac{1}{C_{11}-C_{12}}],\\ S_{12}=\frac{1}{2}[\frac{C_{33}}{C_{33}\left(C_{11}+C_{12}\right)-2C_{13}^{2}}-\frac{1}{C_{11}-C_{12}}],\\ S_{13}=-\frac{C_{13}}{C_{33}\left(C_{11}+C_{12}\right)-2C_{13}^{2}},\;\;S_{33}=\frac{C_{11}+C_{12}}{C_{33}\left(C_{11}+C_{12}\right)-2C_{13}^{2}} \end{array}$$

**Figure 8:**
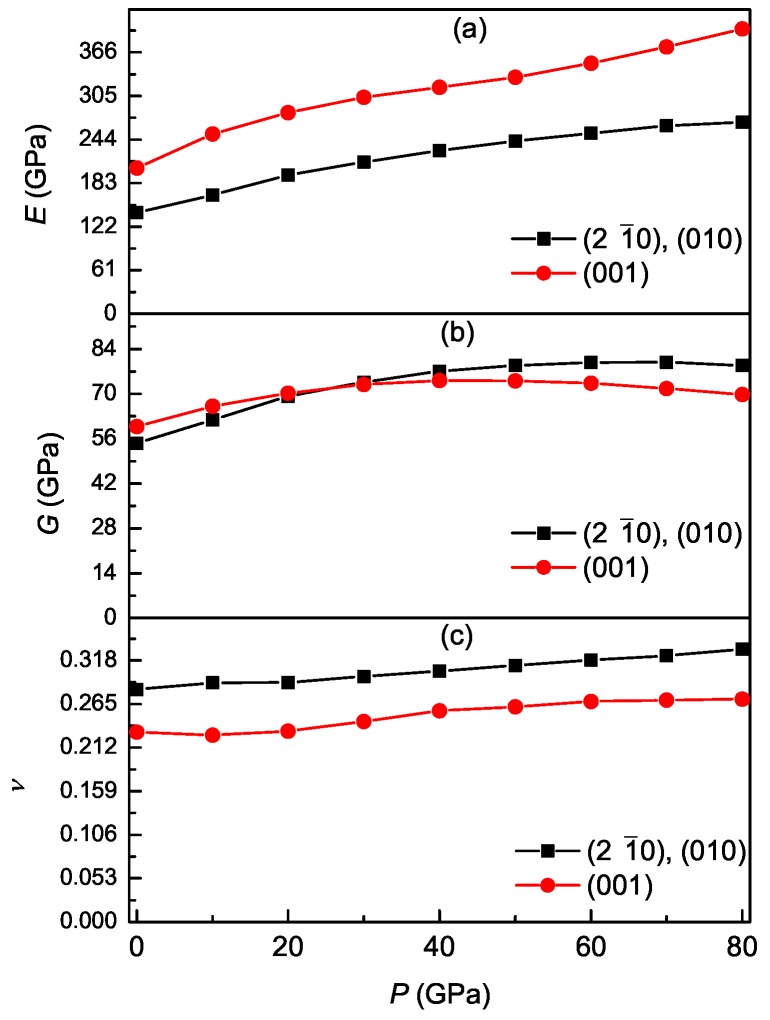
Young’s modulus (**a**), shear modulus (**b**), and Poisson’s ratio (**c**) on the $\left(2\bar{1}0\right)$, (010), and (001) planes as a function of pressure for Ti${}_{3}$Al.

The calculated results of $S_{11}$, $S_{12}$, $S_{13}$, $S_{33}$, and $S_{44}$ for Ti${}_{3}$Al under different pressures are presented in Table 3. The calculated results of Young’s modulus, shear modulus, and Poisson’s ratio on the prismatic and basal planes for Ti${}_{3}$Al under different pressures are presented in Figure 8. It is clearly found that the values of $E_{\left(2\bar{1}0\right)}$, $E_{\left(010\right)}$, and $E_{\left(001\right)}$ on the prismatic and basal planes increase with pressure. The Young’s modulus on the $\left(2\bar{1}0\right)$ and (010) prismatic planes are much smaller than the counterpart on the (001) basal plane, indicating the very significant anisotropic behavior of Ti${}_{3}$Al under different pressures. Meanwhile, the difference between the prismatic planes $E_{\left(2\bar{1}0\right)}$ and $E_{\left(010\right)}$ and the basal plane $E_{\left(001\right)}$ increases with pressure, indicating that the elastic anisotropy of Ti${}_{3}$Al increases with pressure. However, the shear modulus on the two prismatic planes are closed to the counterpart on the basal plane, and the same result occurs for Poisson’s ratio.

An alternative method is the investigation of various anisotropic factors. The shear anisotropic factor for the {100} planes between 〈011〉 and 〈010〉 directions is [31]

(17) $$\begin{array}{c} A_{\left\{100\right\}}=\frac{4C_{44}}{C_{11}+C_{33}-2C_{13}} \end{array}$$

For the {010} planes between 〈211〉 and 〈001〉 directions, it is [31]

(18) $$\begin{array}{c} A_{\left\{010\right\}}=\frac{4C_{55}}{C_{22}+C_{33}-2C_{23}} \end{array}$$

For the {001} planes between 〈110〉 and 〈120〉 directions, it is [31]

(19) $$\begin{array}{c} A_{\left\{001\right\}}=\frac{4C_{66}}{C_{11}+C_{22}-2C_{12}} \end{array}$$

**Figure 9:**
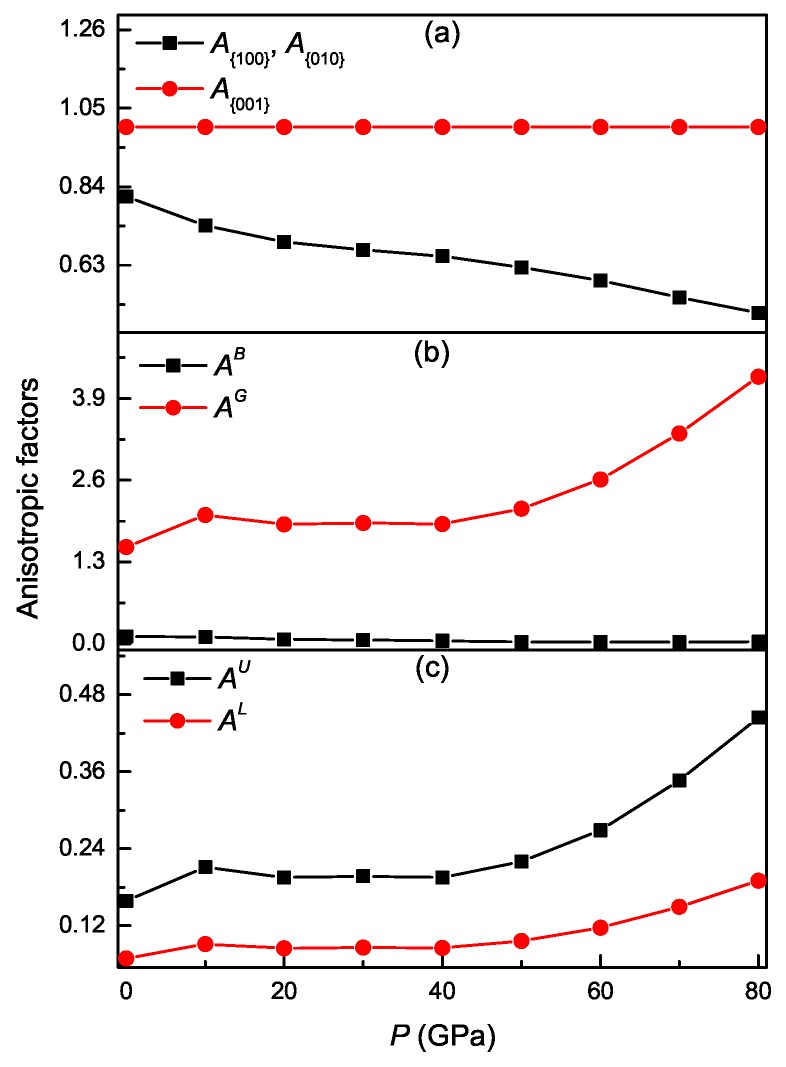
Shear anisotropic factors $A_{\left\{100\right\}}$, $A_{\left\{010\right\}}$, and $A_{\left\{001\right\}}$ (**a**), percentage anisotropy in compressibility $A^{B}$ and shear $A^{G}$ (**b**), the universal anisotropy index $A^{U}$ and the log-Euclidean anisotropy index $A^{L}$) (**c**) as a function of pressure for Ti${}_{3}$Al.

For an isotropic material, the value of $A_{\left\{100\right\}}$, $A_{\left\{010\right\}}$, and $A_{\left\{001\right\}}$ must be unity. A deviation less than or greater than unity represents the degree of anisotropy. The calculated results of $A_{\left\{100\right\}}$, $A_{\left\{010\right\}}$, and $A_{\left\{001\right\}}$ for Ti${}_{3}$Al under different pressures are presented in Figure 9. It is clearly seen that the values of $A_{\left\{100\right\}}$ and $A_{\left\{010\right\}}$ are significantly smaller than unity and decrease monotonously with pressure, whereas the value of $A_{\left\{001\right\}}$ under different pressures is always equal to unity. These results show that as pressure increases, the elastic anisotropy on the {100} and {010} planes of Ti${}_{3}$Al increases, while the compound always exhibits isotropic behavior on the {001} basal plane, which agrees with the general property of hexagonal materials.

The percentage anisotropy for the bulk modulus ($A^{B}$) and shear modulus ($A^{G}$) is given by [40]

(20) $$\begin{array}{c} A^{B}=\frac{B_{V}-B_{R}}{B_{V}+B_{R}}\times 100\%,\;\;A^{G}=\frac{G_{V}-G_{R}}{G_{V}+G_{R}}\times 100\% \end{array}$$

The universal anisotropy index ($A^{U}$) is given by [41]

(21) $$\begin{array}{c} A^{U}=\frac{B_{V}}{B_{R}}+5\frac{G_{V}}{G_{R}}-6 \end{array}$$

The scalar log-Euclidean anisotropy index ($A^{L}$) is given by [42]

(22) $$\begin{array}{c} A^{L}=\sqrt{{\left[\ln\left(\frac{B_{V}}{B_{R}}\right)\right]}^{2}+5{\left[\ln\left(\frac{G_{V}}{G_{R}}\right)\right]}^{2}} \end{array}$$

For an isotropic material, the values of $A^{B}$, $A^{G}$, $A^{U}$, and $A^{L}$ must be zero. A deviation greater than zero represents the degree of anisotropy. The calculated results of $A^{B}$, $A^{G}$, $A^{U}$, and $A^{L}$ are also presented in Figure 9 for Ti${}_{3}$Al under different pressures. It is clearly seen that as pressure increases, the value of $A^{B}$ is almost kept at zero, while that of $A^{G}$ increases approximately. At identical pressure, the value of $A^{B}$ is significantly smaller than that of $A^{G}$. Similar to $A^{G}$, the values of $A^{U}$ and $A^{L}$ also increase approximately with pressure. The results indicate that the shear anisotropy of Ti${}_{3}$Al is more significant than the compressibility anisotropy, and its elastic anisotropy is approximately enhanced with pressure.

### 3.5. Pressure-Dependent Acoustic and Related Properties

The pure longitudinal ($v_{l}$) and transverse ($v_{t}$) sound velocities in the [100] and [001] principal directions for hexagonal Ti${}_{3}$Al can be calculated from the obtained constants $C_{ij}$s of a single crystal following the procedure of Brugger [43]. The sound velocities in the [100] direction are given by [44,45]

(23) $$\begin{array}{c} \left[100\right]v_{l}=\sqrt{\frac{C_{11}-C_{12}}{2\rho }},\;\;\left[010\right]v_{t1}=\sqrt{\frac{C_{11}}{\rho }},\;\;\left[001\right]v_{t2}=\sqrt{\frac{C_{44}}{\rho }} \end{array}$$

and those in the [001] direction are given by [44,45]

(24) $$\begin{array}{c} \left[001\right]v_{l}=\sqrt{\frac{C_{33}}{\rho }},\;\;\left[100\right]v_{t1}=\left[010\right]v_{t2}=\sqrt{\frac{C_{44}}{\rho }} \end{array}$$

where $v_{t_{1}}$ and $v_{t_{2}}$ correspond to the first and the second transverse modes, and ρ represents the mass density of the compound. Since these sound velocities are determined by the elastic constants, their anisotropic properties also reflect the elastic anisotropy in Ti${}_{3}$Al. Additionally, the longitudinal ($V_{L}$) and transverse ($V_{T}$) sound velocities of polycrystal Ti${}_{3}$Al can also be calculated from the obtained bulk modulus *B* and shear modulus *G*, which are given by [46]

(25) $$\begin{array}{c} V_{L}=\sqrt{\frac{3B+4G}{3\rho }},\;\;V_{T}=\sqrt{\frac{G}{\rho }} \end{array}$$

Further, the average sound velocity ($V_{M}$) can be calculated by [47]

(26) $$\begin{array}{c} V_{M}={\left[\frac{1}{3}\left(\frac{1}{V_{L}^{3}}+\frac{2}{V_{T}^{3}}\right)\right]}^{-\frac{1}{3}} \end{array}$$

**Figure 10:**
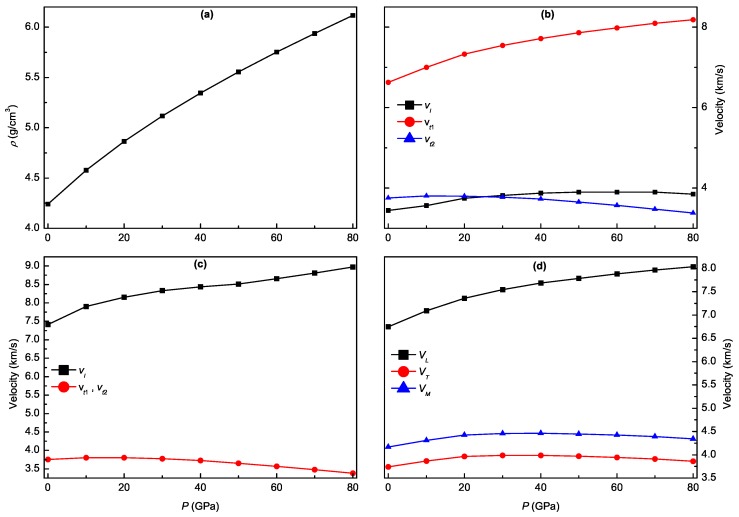
Mass density ρ (**a**), longitudinal and transverse sound velocities $v_{l}$, $v_{t1}$, and $v_{t2}$ in the [100] (**b**) and [001] (**c**) principle directions, and polycrystalline longitudinal, transverse, and average sound velocities $V_{L}$, $V_{T}$, and $V_{M}$ (**d**) as a function of pressure for Ti${}_{3}$Al.

The calculated results of longitudinal and transverse sound velocities for Ti${}_{3}$Al under different pressures are presented in Figure 10. It is clearly seen that the values of $\left[100\right]v_{l}$, $\left[010\right]v_{t1}$, and $\left[001\right]v_{l}$ of a single crystal increase while those of $\left[001\right]v_{t2}$, $\left[100\right]v_{t1}$, and $\left[010\right]v_{t2}$ decrease with pressure, which is consistent with the variation trend of the corresponding elastic constants. Meanwhile, the value of $V_{L}$ of the polycrystal also increases while those of $V_{T}$ and $V_{M}$ increase firstly and then decrease with pressure. Moreover, the value of $\left[100\right]v_{l}$ is much smaller than that of $\left[001\right]v_{l}$ at identical pressure, and the corresponding absolute difference increases with pressure. The results indicate that the anisotropy of sound velocity for Ti${}_{3}$Al is very significant and increases with pressure.

The minimum thermal conductivity is defined as an extreme value of the thermal conductivity decreasing with temperature. From the obtained Young’s modulus and mass density, the minimum thermal conductivity ($k_{min}$) of Ti${}_{3}$Al can be calculated according to Clarke’s model [48]:

(27) $$\begin{array}{c} k_{min}=0.87k_{B}\sqrt[{3}]{{\left(\frac{n\rho N_{A}}{M}\right)}^{2}}\sqrt{\frac{E}{\rho }} \end{array}$$

where $k_{B}$, *n*, $N_{A}$, and *M* are, in turn, the Boltzmann constant, total atoms per primitive cell, Avogadro’s number, and relative molecular weight. However, the model averages the anisotropic elastic stiffness of a crystal. To calculate precisely the minimum thermal conductivity of the crystal with elastic anisotropy, the model was further modified by Liu et al. [49] as

(28) $$\begin{array}{c} k_{min}={\left\{\frac{1}{3}\left[2{\left(\frac{1}{2+2\nu }\right)}^{-\frac{3}{2}}+{\left(\frac{1}{3-6\nu }+\frac{2}{3+3\nu }\right)}^{-\frac{3}{2}}\right]\right\}}^{-\frac{1}{3}}k_{B}\sqrt[{3}]{{\left(\frac{n\rho N_{A}}{M}\right)}^{2}}\sqrt{\frac{E}{\rho }} \end{array}$$

**Figure 11:**
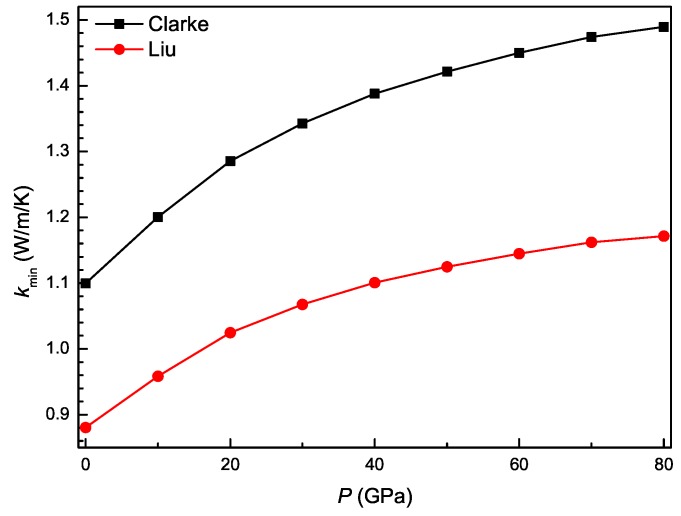
Minimum thermal conductivities as a function of pressure for Ti${}_{3}$Al.

Figure 11 shows the pressure dependence of minimum thermal conductivity for Ti${}_{3}$Al. It is clearly observed that the value of $k_{min}$ obtained from the two models increases monotonously with pressure. At the same pressure, the $k_{min}$ value calculated by the modified Clarke’s model is significantly smaller than that calculated by Clarke’s model. This shows that the minimum thermal conductivity of Ti${}_{3}$Al is markedly reduced after considering its elastic anisotropy.

**Figure 12:**
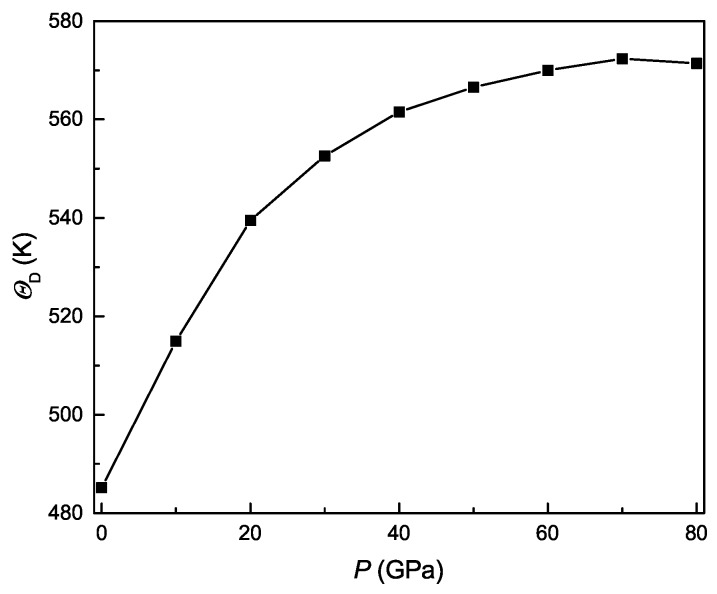
Debye temperature as a function of pressure for Ti${}_{3}$Al.

In addition to minimum thermal conductivity, the Debye temperature ($\Theta_{D}$) of Ti${}_{3}$Al can be calculated from the average sound velocity of its polycrystal by [47]

(29) $$\begin{array}{c} \Theta_{D}=\frac{h}{k_{B}}{\left[\frac{3n}{4\pi }\left(\frac{N_{A}\rho }{M}\right)\right]}^{\frac{1}{3}}V_{M} \end{array}$$

where the parameters *h*, $k_{B}$, *n*, $N_{A}$, and *M* are, in turn, the Plank constant, the Boltzmann constant, number of atoms in the molecule formula, Avogadro’s number, and molecular weight. Figure 12 show the pressure dependence of Debye temperature for Ti${}_{3}$Al. It is clearly found that the value of $\Theta_{D}$ increases monotonously with pressure and exhibits a change trend similar to the minimum thermal conductivity, following the Callaway–Debye theory [50]. Experimentally, the Debye temperature of Ti${}_{3}$Al was determined as 495 K from specific heat measurements [51] and 478 K from the single-crystal X-ray diffraction pattern [52], which agree well with our calculated value of 485.16 K at zero pressure.

## 4. Conclusions

The elastic constants and their related properties, such as elastic moduli, Poisson’s ratio, anisotropic factor, acoustic velocity, minimum thermal conductivity, and Debye temperature, were investigated for hexagonal Ti${}_{3}$Al under different pressures up to 80 GPa by using first-principles calculations. The present results at zero pressure are in good agreement with the previous experimental and theoretical values. The resistance to compression along the *c* axis is better than along the *a* axis under each pressure. According to the mechanical stability criteria under isotropic pressure, the hexagonal structure of Ti${}_{3}$Al is judged to be mechanically stable under pressures up to 71.4 GPa. The bulk modulus, shear modulus, Young’s modulus, Poisson’s ratio, Cauchy pressures, and hardness were calculated, which show that the ductility of Ti${}_{3}$Al is improved with pressure. The linear bulk modulus, the direction-dependent Young’s modulus, shear modulus, and Poisson’s ratio, the shear anisotropic factors, the percentage anisotropy of bulk modulus and shear modulus, and the universal and the log-Euclidean anisotropy indexes were also calculated, which show that the elastic anisotropy of Ti${}_{3}$Al is very significant and increases with pressure. Moreover, the significant anisotropy of sound velocity for Ti${}_{3}$Al increases with pressure. The obtained minimum thermal conductivity and Debye temperature increase with pressure.

## Figures and Tables

**Table 1:** The optimized lattice parameters *a* (in Å), *c*/*a*, and elastic constants (in GPa) of Ti${}_{3}$Al at zero pressure.

	*a*	c/a	$C_{11}$	$C_{12}$	$C_{13}$	$C_{33}$	$C_{44}$
Present	5.758	0.808	186.18	85.57	62.76	233.03	59.75
Exp. [21,26]	5.77	0.80	183.2	89.0	62.6	225.1	64.1
Theo. [22]	5.74	0.81	184.67	82.37	63.41	225.08	53.97
Theo. [24]	5.772	0.803					72
Theo. [9]	5.76	0.809	192.2	80.5	62.5	232.9	61.6
Theo. [23]	5.72	0.81	185	83	63	231	57
Theo. [25]	5.64	0.81	221	71	85	238	69

**Table 2:** The elastic constants $C_{ij}$ (in GPa) of Ti${}_{3}$Al under pressure up to 80 GPa.

*P*	$C_{11}$	$C_{12}$	$C_{13}$	$C_{33}$	$C_{44}$
0	186.18	85.57	62.76	233.03	59.75
10	224.30	108.15	75.51	286.13	66.15
20	261.44	124.83	89.61	323.41	70.22
30	291.09	142.23	105.72	354.83	72.90
40	318.32	157.86	122.34	380.17	74.16
50	343.06	174.27	135.46	402.06	74.06
60	366.48	191.39	149.52	430.75	73.29
70	389.37	208.75	161.36	460.66	71.69
80	409.54	228.60	172.83	492.29	69.81

**Table 3:** The elastic compliances $S_{ij}$ (in $\times 10^{-3}$GPa${}^{-1}$) of Ti${}_{3}$Al under pressure up to 80 GPa.

*P*	$S_{11}$	$S_{12}$	$S_{13}$	$S_{33}$	$S_{44}$
0	7.07	−2.87	−1.13	4.9	16.74
10	6.01	−2.6	−0.901945	3.97	15.12
20	5.15	−2.17	−0.823186	3.55	14.24
30	4.71	−2.01	−0.804515	3.3	13.72
40	4.37	−1.86	−0.809706	3.15	13.48
50	4.14	−1.79	−0.790763	3.02	13.5
60	3.96	−1.75	−0.764448	2.85	13.64
70	3.80	−1.74	−0.722140	2.68	13.95
80	3.73	−1.8	−0.679304	2.51	14.33
